# Notes on Preparations for the Sick

**Published:** 1903-03

**Authors:** 


### Notes on Preparations for the Sick.

**Kefir and Kefir Powders.**—AYLESBURY DAIRY COMPANY, London.—Kefir is a product of the fermentation of milk which has undergone a preliminary sterilization by special ferments derived from Kefir grains. Careful selection of the organisms present in the grains has resulted in the preparation of a ferment which produces lactic acid fermentation from milk sugar, and has a slight proteolysing action on the proteids. The resulting beverage is thick from the precipitation of the casein in a very finely divided form, and foams slightly from the presence of carbonic acid. The mean composition of Kefir is as follows:—

| | | |
|---|---|---|
| Water (including a little $CO_2$) ... | 89.99 | per cent. |
| Alcohol ... ... ... ... ... ... | 0.70 | ,, |
| Lactic Acid ... ... ... ... ... | 0.73 | ,, |
| Fat ... ... ... ... ... ... ... | 2.05 | ,, |
| Sugar ... ... ... ... ... ... | 2.40 | ,, |
| Casein ... ... ... ... ... ... | 2.90 | ,, |
| Albumin ... ... ... ... ... ... | 0.21 | ,, |
| Albumoses ... ... ... ... ... | 0.30 | ,, |
| Mineral Matter ... ... ... ... | 0.72 | ,, |

In the mouth the pleasantly acid taste, due to the lactic acid, stimulates the secretory glands and promotes an increased flow of saliva, preventing the mouth from becoming dry. In the stomach both the lactic acid and alcohol act as gastric stimulants; the casein being already precipitated in small masses, and separated from the salts with which it is combined in milk, cannot be curdled by the ferment of the stomach, and therefore there is no formation of compact masses, which pass undigested through the intestines, as with milk. As the casein contains all the organic phosphorus, and most of the iron and sulphur, it is seen that milk does not provide sufficient nourishment in this respect; the use of Kefir, on the other hand, supplies these important constituents, and this explains its value in chlorosis, anæmia, scrofulous disease, in convalescence, and in diseases where malnutrition of tissue occurs.

The powders are used for the preparation of Kefir at home. A pint of milk is raised to boiling point in a saucepan, and rapidly cooled to 70° F. by standing it in a convenient vessel in cold water; one powder is dissolved by stirring or shaking in the milk. The mixture is poured into a clean champagne bottle, which is securely corked, and kept on its side at a temperature of from 60° to 70° F. The bottle must be gently shaken two or three times a day, and in two to three days the Kefir is ready for consumption. If kept on ice, after the third day the Kefir can be preserved in good condition for a week or more.

---

**Calcusol.**—Alfred Bishop, Limited, London.—This is a granular effervescent piperidine para-sulphamine benzoate in combination with bicarbonate of potash, in proportion of ten grains of the latter with five of the former in each drachm dose. It should be of use in the multiple and varied conditions which may be traced to uric acid. This salt of piperidine has the composition, $SO_2\ NH_2\ C_6\ H_4\ COOH\ C_5\ H_{11}\ N$. It is neutral, freely soluble in water, and not toxic even in large quantities. It appears to be the best preparation yet devised for the solution of uric acid depositions, whether in the joints or elsewhere.

---

**Petanelle.**—Paté, Burke, & Co., 6 Wool Exchange, London.—This preparation of fibrous peat appears to be of great value to those who suffer from perspiring and malodorous feet, inasmuch as the Petanelle foot-powder has proved itself to be of use for the relief of overheated and tender feet. It is deodorant, antiseptic, and absorbent. A little in the boot is sufficient, as it is not necessary that the powder should be in immediate contact with the skin. Petanelle obtained the Grand Prix at Ostend, 1901.

